# No Change in Medial Olivocochlear Efferent Activity during an Auditory or Visual Task: Dual Evidence from Otoacoustic Emissions and Event-Related Potentials

**DOI:** 10.3390/brainsci10110894

**Published:** 2020-11-23

**Authors:** W. Wiktor Jedrzejczak, Rafal Milner, Malgorzata Ganc, Edyta Pilka, Henryk Skarzynski

**Affiliations:** 1 Institute of Physiology and Pathology of Hearing, ul. M. Mochnackiego 10, 02-042 Warsaw, Poland; rafal.milner@gmail.com (R.M.); m.ganc@ifps.org.pl (M.G.); e.pilka@ifps.org.pl (E.P.); skarzynski.henryk@ifps.org.pl (H.S.); 2 World Hearing Center, ul. Mokra 17, 05-830 Nadarzyn, Poland

**Keywords:** attention, medial olivocochlear efferent system, otoacoustic emissions, contralateral acoustic stimulation, event-related potentials, P3, EEG

## Abstract

The medial olivocochlear (MOC) system is thought to be responsible for modulation of peripheral hearing through descending (efferent) pathways. This study investigated the connection between peripheral hearing function and conscious attention during two different modality tasks, auditory and visual. Peripheral hearing function was evaluated by analyzing the amount of suppression of otoacoustic emissions (OAEs) by contralateral acoustic stimulation (CAS), a well-known effect of the MOC. Simultaneously, attention was evaluated by event-related potentials (ERPs). Although the ERPs showed clear differences in processing of auditory and visual tasks, there were no differences in the levels of OAE suppression. We also analyzed OAEs for the highest magnitude resonant mode signal detected by the matching pursuit method, but again did not find a significant effect of task, and no difference in noise level or number of rejected trials. However, for auditory tasks, the amplitude of the P3 cognitive wave negatively correlated with the level of OAE suppression. We conclude that there seems to be no change in MOC function when performing different modality tasks, although the cortex still remains able to modulate some aspects of MOC activity.

## 1. Introduction

Everyone with normal hearing sometimes experiences, when focusing on visual information, failure to hear surrounding sounds, for example, while reading we ignore background sounds and may not even hear someone talking to us. Conversely, if we focus our attention on auditory information, such as listening to a radio or another person, then, it is difficult to read. The question is whether such switches of attention are performed only at the cortical level or whether peripheral parts, such as the ear, are also involved.

If the ear itself plays an active or “conscious” part in switching attention, it is most likely to be through activity of the medial olivocochlear (MOC) system. MOC neurons descend from the brainstem to the cochleae of both ears, while the brainstem also receives input from auditory cortex. The function of this system is not entirely understood; probably its role relates to bilateral hearing tasks such as localization or detecting speech in noise [1,2]. Usefully, the activity of this system can be studied noninvasively by measuring changes in otoacoustic emissions (OAEs) in response to contralateral acoustic stimulation (CAS). With CAS, the amplitude of OAEs decreases, an effect called OAE suppression, OAE inhibition, or the MOC reflex (reviewed in [3,4,5]).

This study concentrates on transiently evoked OAEs (TEOAEs), which are signals recorded up to 20 ms after onset of a stimulus, which is most often a click [6]. Some early TEOAE papers claimed to find there was an effect of attention on TEOAEs even when contralateral stimulation was not involved (e.g., [7]). These experiments were repeated by Michie et al. [8] who found it was not possible to replicate these attentional effects. Some years later, several papers revived interest in the question of whether TEOAEs may in fact depend on attention but this time they involved TEOAE suppression. For example, de Boer and Thornton [9] showed that performing a task did affect measured levels of suppression. Other studies also shown that there was a greater level of OAE suppression during active listening as compared with passive listening (e.g., [10]). However, it is important to note that the suppression findings have been far from conclusive and even the authors acknowledge that the changes are very small and border on insignificance. 

There are also encouraging results from animal studies. For example, Delano et al. [11] showed a decrease in cochlear sensitivity during periods when chinchillas were attending to visual stimuli as compared with when they attended to auditory stimuli. Terreros et al. [12] also showed that the MOC system of mice helped the animals ignore auditory distractors during visual attention. However, it is still unknown whether observations in animals mimic those in humans.

The rationale for the present study was to combine TEOAE measurements with event-related potentials (ERPs) to investigate the effect of switching attention from the visual to the auditory modality. Such an approach has the potential to give insight into whether there is a link between peripheral function (as assessed by TEOAEs) and conscious attention to a particular task (as reflected by the amplitude of the P3 cognitive wave in an ERP measurement). Until now, such an approach has not been used in studies comparing different modalities [9,13,14,15].

The purpose of this study was to investigate whether there was a difference in TEOAE suppression between tasks involving auditory attention on the one hand and visual attention on the other. ERPs were used as an objective measure of attention, while the OAE measurements were being made. TEOAE suppression was evaluated in different windows, and for its highest energy component. An additional perspective was to check whether task performance was affected by noise levels or number of rejected trials, since earlier studies have reported that, curiously, noise seemed to play an important role (e.g., [9,14]).

## 2. Materials and Methods

### 2.1. Experimental Design

ERPs were used to objectively gauge the subject’s state of attention, while TEOAEs in response to clicks were used to gauge inner ear function. As a test of descending neural activity, TEOAE levels were evaluated with and without CAS by broadband noise, a paradigm known to activate the MOC pathway. 

**Figure 1:**
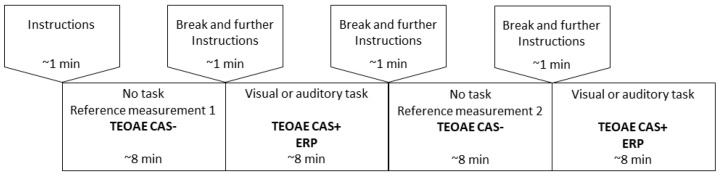
Schematic of measurement setup. TEOAE, transiently evoked otoacoustic emission; CAS+, contralateral acoustic stimulation on; CAS-, contralateral acoustic stimulation off; ERP, event-related potential.

The experiment was based on two attentional tasks, one auditory and the other visual. A schematic of the paradigm is shown in Figure 1. There were four parts to the experiment. First, a reference measurement was made of TEOAEs without CAS, and without any tasks. Second, we introduced a task (randomly auditory or visual) while measuring TEOAEs with CAS. Third, we made a second reference measurement of TEOAEs without CAS. Fourth, we introduced a second task (again auditory or visual at random) while measuring TEOAEs with CAS. During both cognitive tasks, the subject received the same auditory and visual stimuli, but was instructed to direct attention to only one task modality and to ignore the other. The performance of tasks was evaluated by ERPs (which were synchronized with the auditory stimuli but not synchronized with the visual stimuli). In this way we expected to see a P3 wave (a marker of cognition) in ERPs during the auditory task and no P3 during the visual task when attention shifted to another modality. 

**Figure 2:**
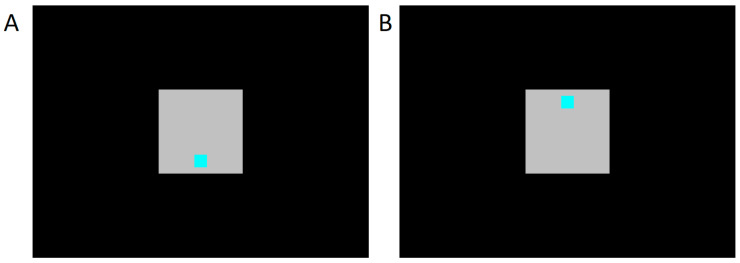
Stimuli used for the visual paradigm as presented on a monitor screen. (**A**) standard; (**B**) Deviant. The background grey field is 10 × 10 cm, the stimulus is an aqua square 1.7 × 1.7 cm.

To manipulate attention, we asked subjects to attend to 5 dB Sound Pressure Level (SPL) decreases in the level of noise presented to the contralateral ear. In the auditory condition, the subject’s task was to tap a button when the noise level decreased (duration 1 s, every 4 ± 2 s). At the same time, the subject was presented with visual stimuli on a computer monitor 1.5 m in front of them. Stimuli were delivered according to a visual odd-ball paradigm (a sequence of standard stimuli randomly interrupted by an inverted deviant stimulus). Standard stimuli were squares shown at the bottom of the screen; deviant stimuli were the same squares but shown at the top (Figure 2). There were 400 (80%) standard stimuli and 100 (20%) deviant stimuli in each recording session. The standard and deviant stimuli were both randomly presented for 100 ms every 2 s. The subjects were seated in such a way that the screen was about 1 m in front of their eyes. 

The tasks were specifically designed to be easy in order to get a clear P3. In preliminary recordings when we used more difficult auditory tasks (1–3 dB SPL changes in CAS), we obtained significantly lower P3 magnitudes. This decrease made it harder to distinguish shifts from auditory to visual attention in ERP recordings. Therefore, we used 5 dB changes in CAS, which are quite easy to pick. The rates of correct responses were similar, with 97% for the auditory task and 96% for the visual task. 

The two parts of the experiment were delivered to subjects in mixed order (in 11 cases the auditory part came first). The whole procedure lasted about 30 min. The duration of recording varied slightly depending on the number of artifacts rejected.

### 2.2. Participants

There were 20 normally hearing adults (age 25–40 years, 14 females) who participated in the study. All subjects had pure tone thresholds better than 25 dB HL at 0.5–8 kHz, normal middle ear function verified by 226 Hz tympanometry (tympanometric peak pressure values between −100 and +100 daPa and peak compensated static acoustic admittance values of 0.2–1.0 mmhos), and no known history of otologic disease. In all subjects, ipsilateral and contralateral middle ear acoustic reflex thresholds (ARTs) for broadband noise were above 80 dB SPL. 

Measurements were initially collected in 24 subjects; however, 4 were later excluded from the analyses due to problems related to the experimental setup, i.e., OAE probe slippage, electrode slippage, or contamination of measurements by noise related to tiredness or movement during the experiment. 

The subjects gave written informed consent prior to participation. The research procedures were approved by the Ethics Committee of the Institute of Physiology and Pathology of Hearing, Poland (approval no. IFPS:KB/09/2015).

### 2.3. TEOAE Procedures

Many OAE studies related to attention use custom-built systems and custom signal acquisition procedures (e.g., [15,16,17]). It is not easy to replicate such experiments or to relate them to those made using other methods. Therefore, one of the underpinnings of this study was to perform experiments that were easy to replicate and which could be easily interpreted by researchers or clinicians using commercial diagnostic equipment. However, we did use different equipment (HearID, Mimosa Acoustics Inc., Champaign, IL, USA) than that used in a previous study (the ILO system, [18]), since the HearID provides better signal-to-noise ratios (SNRs) (e.g., [19]). Higher SNRs facilitate detection of smaller MOC effects (e.g., [20]).

Using the HearID system, TEOAEs were recorded using 65 dB SPL clicks (linear mode). All recordings were performed in a 20 ms acquisition window. TEOAEs were collected for each part of the experiment (Figure 1); each subaverage took about 8 min to collect. To minimize stimulus artifacts the initial 2.5 ms of all responses was windowed out automatically by the system. Responses were filtered in a 0.5–4.5 kHz range and broadband response levels were used for analysis. The quality of recordings was evaluated in terms of broadband SNR, calculated by subtracting the noise levels (in dB) from the response levels (in dB). For MOC reflex studies, SNRs need to be higher than for standard TEOAE studies (e.g., [21]), and therefore, here, all analyzed recordings (with or without CAS) were required to have an SNR of at least 12 dB, as opposed to the usual 3 or 6 dB. 

Recordings were made with and without 60 dB SPL white noise delivered to the contralateral ear. Only the right ear of every subject was tested for TEOAEs. Contralateral noise was delivered to the left ear through an Interacoustics AC40 audiometer (Denmark); its level was 60 dB SPL with 5 dB SPL decreases as described earlier. The sequence of decreases in noise amplitude was controlled by Presentation software (version 16.4).

TEOAE suppression was calculated by two methods. First, by subtracting the response levels with contralateral stimulation from the levels without, i.e., the response level of TEOAEs measured while performing a task was subtracted from the average response level measured during reference measurements 1 and 2. The second method takes into account phase effects and is based on the percentage change in the time domain waveform [22,23]:

(1) $$\Delta_{TEOAE}=100\;\times \;\sqrt{\frac{1}{N}\sum_{n=1}^{N}{\left(a_{quiet}\left[n\right]-a_{noise}\left[n\right]\right)}^{2}}\;/\sqrt{\frac{1}{N}\sum_{n=1}^{N}{\left(a_{quiet}\left[n\right]\right)}^{2}\;},$$

where *N* is the number of samples, *a_quiet_* is the amplitude of the TEOAE waveform measured without CAS (average of reference measurements 1 and 2), and *a_noise_* is the amplitude of the TEOAE waveform measured with CAS (during a task). 

### 2.4. Event-Related Potentials (ERP) Procedures

ERPs were used to objectively gauge the subject’s state of attention during TEOAE recordings. ERPs were obtained from EEG signals recorded by a 32-channel EEG system (Brain Products, Gilching, GmBH, Germany). During the EEG measurement, 24 recording electrodes were used, made up of 22 active electrodes, 1 reference electrode, and 1 ground electrode [24]. Active electrodes were mounted in an EEG cap placed on the head of the subject. The positions of 20 of them accorded with the 10/20 standard, and two electrodes were placed on the mastoids (TP9, TP10). The reference electrode was at FCz and the ground electrode was at AFz. During EEG acquisition, the impedance was monitored and always kept below 10 kΩ for all electrodes. The sampling frequency for each channel was 1000 Hz.

After recording, the EEG signal was analyzed offline using Brain Vision Analyzer 2.2 software (Brain Products, GmBH, Gilching, Germany). In the first step, the signal was re-referenced against the signal recorded from the mastoids. It was also digitally high- and low-pass filtered within the range 0.3–30 Hz. Next, eye-blink artifacts were corrected by zeroing the activation curves of individual independent component analysis (ICA) components corresponding to eye blinks [25,26]. The EEG signal was manually inspected and all artifacts related to muscle activity were removed. The artifact-free EEG signals were used to calculate ERPs. The signal was cut to an epoch of 1200 ms, from 200 ms pre-stimulus to 1000 ms post-stimulus. Subsequently, the level of each epoch was corrected relative to the baseline. Each of the epochs processed in this way was, then, assessed again in terms of artifacts.

The final step was to obtain average ERPs from the previously prepared and artifact-free epochs. The two ERP averages were calculated for each subject, i.e., one from the EEG signal recorded during the auditory task, and a second during the visual task. Although we recorded the EEG signal from 22 active electrodes, ERPs were analyzed only for the following three positions along the midline: Fz, Cz, and Pz. In the present study, we simply wanted to identify the P3 wave and since P3 has frontal and parietal generators, we chose the midline electrodes.

The P3 wave in signals recorded during an auditory task was defined as the largest positive wave in the 250–600 ms window. The N1 and P2 waves in the auditory and visual tasks were defined as the maximum negative peak in the 90–170 ms and 170–250 ms windows respectively. The semi-automatic algorithm in Brain Analyzer software was used to detect the peaks in each of the listed waves for each subject. 

### 2.5. Data Analysis: Matching Pursuit

For extracting the main components of the signal, a method based on the matching pursuit (MP) algorithm was used [27]. The MP method allows a TEOAE signal to be decomposed into waveforms of defined frequency, latency, duration, and amplitude. The method fits the characteristics of TEOAE signals well, and is able to pick ”resonance modes” of TEOAEs (i.e., the most prominent waveform within a TEOAE signal), and show its suppression [28,29].

**Figure 3:**
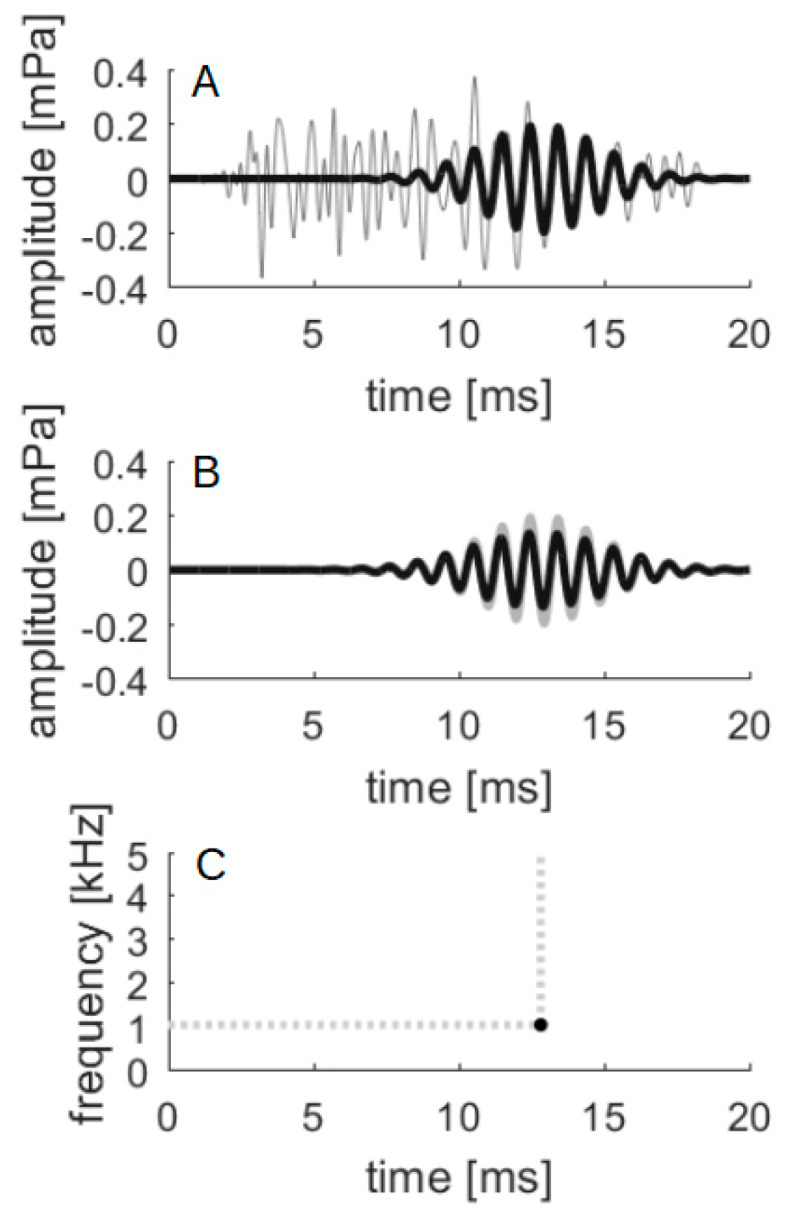
Example of application of matching pursuit. (**A**) TEOAE signal without CAS (grey) with the strongest resonant mode (superimposed, black) detected by matching pursuit, the highest energy single-frequency component of the TEOAE; (**B**) The same resonant mode (grey) with superimposed mode obtained from TEOAE with CAS (black), the amplitude is now slightly decreased; (**C**) The point in the time–frequency plane where the resonant mode has maximum amplitude.

An example of applying the MP algorithm to calculate TEOAE suppression is shown in Figure 3. In the top panel is a typical TEOAE signal (grey) with the highest energy resonant mode picked up by the MP method superimposed (black). In the middle panel, this resonant mode is shown with and without CAS. The bottom panel shows the time–frequency position of this resonant mode. The whole TEOAE signal is composed from several (usually about 20) resonant modes which span from around 4 kHz and 4 ms to around 1 kHz and 15 ms (e.g., [30]).

### 2.6. Statistical Analysis

All analyses were made in Matlab (version 2020a, MathWorks, Natick, MA, USA). All datasets had normal distributions as indicated by a Shapiro–Wilk test. The statistical significance of the mean difference between groups was evaluated for all parameters using a *t*-test or ANOVA. For some analyses, Pearson correlations were calculated. In all analyses, a 95% confidence level (*p* < 0.05) was chosen as the criterion of significance.

## 3. Results

### 3.1. ERPs

**Figure 4:**
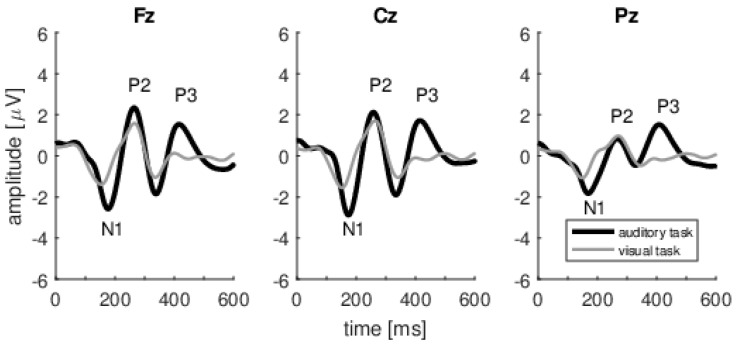
Grand-average ERPs for all subjects recorded during the auditory task (black lines) and the visual task (grey lines). In both tasks the ERPs are synchronized to auditory stimuli. The consecutive plots show ERPs at Fz, Cz, and Pz electrodes, respectively. P3 is evident in responses to stimuli during the auditory task but, because of the synchronization arrangement, seems to be absent during the visual task.

States of attention during performance of auditory and visual tasks were verified objectively by the presence or absence of ERPs. That is, we verified that in each subject, in response to the instruction to pay attention and count the auditory stimuli, the P3 wave, an electrophysiological marker of cognitive processes [30,31], was present. Alternatively, when the subject performed a visual task (while auditory stimuli were delivered in the same way as in the auditory task) there was no clear P3 wave (synchronized to the auditory stimuli), indicating a switch of attention from auditory to visual mode. Figure 4 shows, for three electrode positions, the grand-average ERPs for all subjects during both auditory and visual tasks, and it is clear that a large P3 wave appears only when the subject was performing an auditory task (thick black lines in Figure 4). N1 and P2 waves are present for both tasks as they are related to preattentive, involuntary reaction of the brain to stimuli. The largest P3 wave is present at the centro-parietal (Cz and Pz) electrodes, which is in line with previous studies of the P3 wave (e.g., [32]) and confirms the location of the P3 generator in parietal brain regions, which are known to be engaged in attentional processes (e.g., [33,34]).

### 3.2. Average TEOAE Analysis

The magnitude of TEOAE suppression by CAS was calculated by subtracting TEOAE response levels measured with CAS from those measured without CAS. It was calculated as a raw dB effect and also as a percentage change.

Additionally, three ways of estimating TEOAE suppression were used in order to check whether significant effects could be detected.

**Figure 5:**
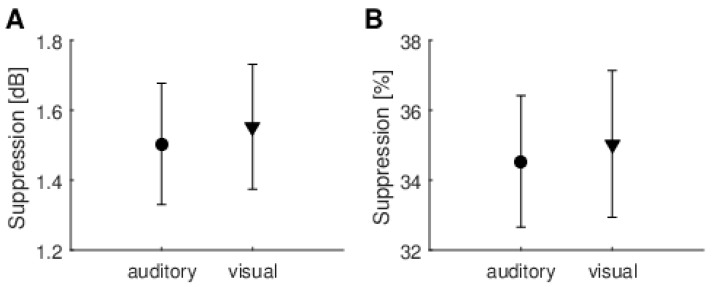
Average suppression levels for TEOAEs measured in a 2.5–20 ms window under auditory and visual attention conditions. (**A**) Suppression expressed in dB; (**B**) Suppression expressed as % change in the signal. Whiskers indicate standard errors. There were no statistically significant differences between any of the data (*p* > 0.05).

The first, and the most commonly used approach to evaluate TEOAE suppression, was to calculate suppression over the whole of the TEOAE signal (2.5–20 ms post-stimulus window), as shown in Figure 5. There was no significant difference between TEOAE suppression measured during auditory and visual tasks (for dB effect, t(38) = −0.19, *p* = 0.84 and for percentage effect, t(38) = −0.18, *p* = 0.85). Average SNRs and their standard deviations for the TEOAEs used to calculate suppressions were as follows: 21 ± 3 dB for reference 1, 21 ± 4 dB for reference 2, 21 ± 4 dB for the auditory task, and 20 ± 4 dB for the visual task.

**Figure 6:**
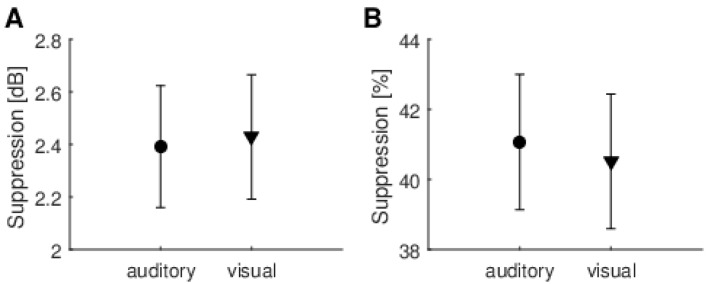
Average suppression levels for TEOAEs measured in an 8–18 ms window under auditory and visual attention conditions. (**A**) Suppression expressed in dB; (**B**) Suppression expressed as % change in the signal. Whiskers indicate standard errors. There were no statistically significant differences between any of the data (*p* > 0.05).

The second approach for evaluating TEOAE suppression was to calculate it for the window over which the MOC reflex is usually highest (8–18 ms post-stimulus window), as shown in Figure 6. This window is also often used in MOC studies (e.g., [10]). By comparing Figure 6 with Figure 5 it can be seen that suppression values increased from around 1.5 dB to 2.4 dB, and from 34% to 41%. Again, there was no significant difference between suppression measured during auditory or visual tasks (for dB effect, t(38) = −0.11, *p* = 0.91 and for percentage effect, t(38) = 0.20, *p* = 0.84). Average SNRs and their standard deviations for the TEOAEs used to calculate suppressions were the following: 20 ± 4 dB for reference 1, 20 ± 4 dB for reference 2, 19 ± 5 dB for the auditory task, and 16 ± 5 dB for the visual task.

**Figure 7:**
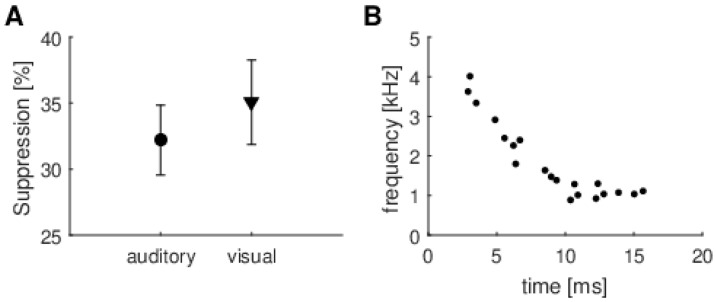
Suppression of resonant modes detected by matching pursuit for auditory and visual attention conditions. (**A**) Average suppression for TEOAE main resonant mode found by matching pursuit as shown in Figure 3. Whiskers indicate standard errors. There were no statistically significant differences between any of the data (*p* > 0.05); (**B**) Time–frequency positions of resonant modes used for calculations.

The third approach was developed specifically for this study. It builds on the idea that attention probably affects only the main components of a signal. The MP method was used to identify the most prominent component of a TEOAE, and then suppression was calculated only for this component (Figure 7). Once more, there was no significant difference between suppression measured during auditory and visual tasks (t(38) = −0.69, *p* = 0.49). Figure 7B shows the time–frequency positions of these main prominent TEOAE components used for later calculations (one point for each subject). The pattern shown by the position of these components is typical for TEOAE, and can be compared with previous studies (e.g., [28]). However, it is noteworthy that for each person the main component has different time–frequency positions (they do not group in any particular frequency or time range).

### 3.3. Individual TEOAEs

**Figure 8:**
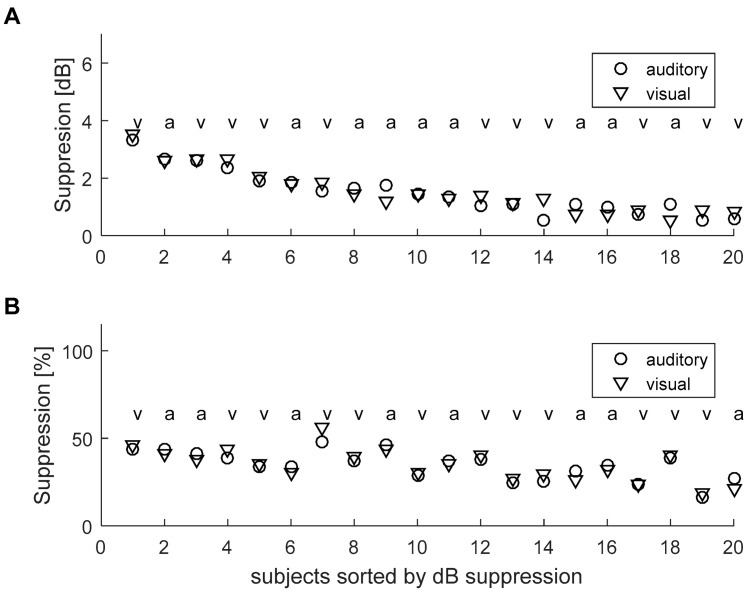
Individual data for all subjects of MOC reflex during visual and auditory tasks, and shown in order of suppression level calculated as raw dB effect. (**A**) Raw dB effect; (**B**) Normalized % effect. Above the results for each subject is a letter indicating which modality gave the higher effect, i.e., a, auditory and v, visual.

There might be situations where changes in TEOAE suppression, even if non-significant, might more often favor a certain modality, i.e., more subjects have greater suppression for that modality. Thus, in Figure 8 we show responses for individual subjects. Here, for nine subjects, suppression was greater for the auditory task and, in 11 subjects, it was greater for the visual task, calculated as raw dB. In terms of percentage change, the number was eight for the auditory task and 12 for the visual task. Since these numbers are nearly equal, we conclude that there does not seem to be a trend.

**Figure 9:**
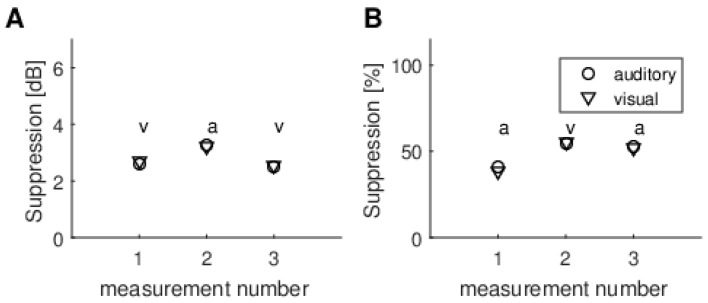
Example of multiple repeats of the experiment in a single subject. (**A**) Raw dB effect; (**B**) Normalized % effect. Above the results for each run is a letter indicating which modality gave the higher effect, i.e., a, auditory and v, visual.

Additionally, we selected one subject and repeated the experiment three times across a three-month period. It might be supposed that if there was a different effect of task modality, even if it were on the border of significance, it would be repeated across different sessions, i.e., for the same person the higher TEOAE suppression would be for the same modality. As can be seen, some subjects had very small suppression, meaning it was hard to distinguish the higher attentional states. Therefore, we selected one subject who had prominent suppression of about 2.5 dB (Subject 3 in Figure 8). The results of three consecutive experiments on this person are shown in Figure 9. It can be seen that, at each session, the suppression is not consistently highest for any particular modality. In fact, different modalities are numerically higher depending on whether the calculation is done using dB or percent suppression. Furthermore it is clear that the differences between the modalities are smaller than the differences between consecutive sessions.

### 3.4. Combined ERP and TEOAE Results

**Figure 10:**
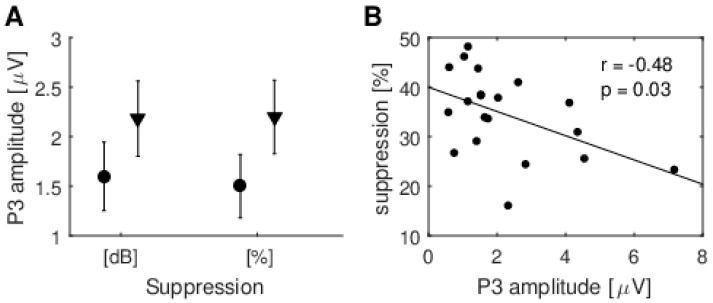
Combined ERP and TEOAE results. (**A**) P3 amplitude (while performing an auditory task) grouped according to whether TEOAE suppression was stronger for that task (circles, average for subjects who had stronger suppression during the auditory task and triangles, average for subjects who had stronger suppression during the visual task). Suppressions are calculated in both dB and % change in signal; (**B**) TEOAE suppression (as a % change in signal) during an auditory task plotted against P3 amplitude at the Cz electrode. P3 amplitude (during the auditory task) negatively correlates with the level of suppression.

Some previous studies have suggested that the MOC system has a significant effect on only one particular modality (e.g., [9,15]). Therefore, we wanted to explore whether subjects that showed high TEOAE suppression in one particular task also had different P3 wave amplitudes between tasks. The subjects were divided into two groups according to whether the suppression (calculated as raw dB or % effect) was higher in the visual task (11 subjects for suppression in dB, 12 subjects for suppression in %, Figure 8) or the auditory task (nine subjects for suppression in dB, eight subjects for suppression in %, Figure 8). The results are shown in Figure 10A, and there is no statistically significant difference (for dB effect t(18) = −1.68, *p* = 0.28 and for percentage effect, t(38) = −1.33, *p* = 0.20).

We also tried to correlate the amplitudes of waves N1, P2, and P3 during TEOAE suppression with TEOAE response level. We found only one significant correlation for Cz position (r = −0.48, *p* = 0.031, while, for Pz, the correlation was very close to significant: r = −0.43, *p* = 0.057). Figure 10B shows the correlation between TEOAE suppression and P3 wave amplitude recorded during an auditory task. It shows how the amplitude of P3 increased as the normalized % suppression decreased. Note that the normalized % calculation incorporates both amplitude and phase of the signal, while the dB measure is based only on amplitude, and given the subtle nature of the observed changes, it is not surprising that a significant correlation was found only for %, not for dB change. In the case of the visual task, we did not record evoked potentials associated with these stimuli, and therefore we could not correlate responses with suppression (furthermore, as explained earlier, there was no P3 wave during the visual task). A significant correlation was found only for the signal analyzed in the 2.5–20 ms window; there were no significant result for the 8–18 ms window or for the dominant resonant modes.

### 3.5. Other Factors

**Figure 11:**
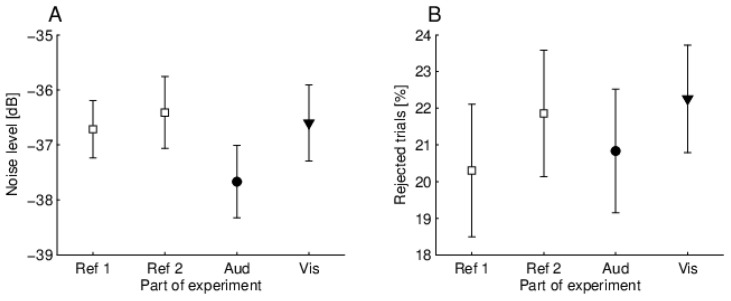
Average noise levels (**A**) and percent of rejected trials (**B**), for four modes, i.e., reference 1 and 2 (squares), auditory (circles), visual (triangles). Ref 1, reference 1; Ref 2, reference 2; Aud, auditory task; Vis, visual task.

We also analyzed other factors that might depend on the quality of the data or might change when a subject performs certain tasks. Some previous studies have indicated that other measurement-related factors might affect the results of MOC studies involving different attentional states, for example, when attending to a difficult task the subject might be more tense, which might then lead to an increase in artifacts [9,16] and could end up affecting noise levels or the number of rejected trials. Figure 11 shows the average TEOAE noise levels and the percent of rejected trials during different parts of an experiment. Figure 11A shows that, although noise levels were slightly lower when an auditory task was performed, repeated measures ANOVA revealed that there were no statistically significant differences (F(3,57) = 2.39, *p* = 0.078). Turning to the percentage of rejected trials, the rejection rate was calculated for each experimental part for each subject (in general, the system automatically rejected single trials if they exceeded 55 dB SPL), and the average results are shown in Figure 11B. Again, although there were some variations, repeated measures ANOVA did not show any statistically significant difference (F(3,57) = 2.69, *p* = 0.054).

## 4. Discussion

This study has investigated whether changing attention from the auditory to the visual modality has an effect on the MOC system (as gauged by the amount of TEOAE suppression produced by CAS). A change of attention was confirmed by a corresponding change in the P3 wave; it was present during auditory stimuli and absent during the visual task (while the same auditory stimuli were being delivered). However, despite a clear change in attention, we were unable to observe any change in the MOC system. At the same time, another interesting and novel observation was made, i.e., that the amplitude of the P3 cognitive potential increases at the same time as the level of TEOAE suppression actually decreases.

This study is a continuation of our previous study which focused only on visual attention [18]. In that work, it was found that there was no effect of visual attention on TEOAE suppression. Some other studies have concluded that auditory attention may have a stronger influence on OAE suppression than does visual attention (e.g., [9]). However, we were not able to see such an effect. This might be because of several factors. Here, we tried to control all parameters, and different modality sessions were identical with the only difference being the switch in modality. Therefore, there were no differences between TEOAEs during different modality tasks and there were no differences in noise levels or number of rejected trials. Some previous studies have claimed to have found such differences, but there is the possibility they were related not to attention but to the experimental setup [9,14]. In fact, Francis et al. [16] gave examples of where there were decreases in physiological noise during attentional parts of the experiments, and they were associated with a reduction in subject motion, not by changes in MOC. Our study indirectly backs up this idea, that is, we do not observe changes in physiological noise when switching from a visual task to an auditory task (in fact we deliberately designed the experiment so that there would not be any change in movement between tasks). Our results are also in line with other recent studies, such as that of Dragicevic et al. [17], who found no change in the amplitude of OAEs during attention tasks, although they did find low frequency oscillations. Indeed, it seems that the attentional effects on OAEs, if they are present, are more subtle than just amplitude or broadband suppression changes. However, our experimental setup did not provide a way to explore effects such as low frequency oscillations, and therefore we were unable to directly confirm this interesting observation. We did try to look for other more subtle changes in the signal, for example, by examining specific components using the MP method, but again we failed to see any systematic changes.

It should be underlined that our study was based on easy tasks. It is possible that MOC effects are switched on only when the difficulty becomes higher. This needs to be resolved by dedicated experiments. Here, our intention was to examine the effect on OAE suppression when switching attention from one modality to the other, and such an approach gives the advantage of easily distinguishing ERP effects. Our starting assumption, based on the literature (e.g., [9,14,15]), was that we would see some effect, at least on averaged data. The final results, which demonstrate no effect, might be a starting point for further experiments involving more difficult tasks.

Another issue is measurement reliability. Looking at Figure 9, which shows multiple recordings from the same subject, it is clear that differences in TEOAE suppression values among modalities are smaller than those between consecutive sessions. It should be underlined that the variability of TEOAE suppression between sessions observed here, i.e., about 1 dB, is not related to this particular measurement setup. Using different equipment, other authors have observed even larger variability (e.g., [35]).

When different methods of calculating the MOC reflex were used (raw dB vs. normalized %), it was often the case that one method produced higher figures than the other. This was especially clear for data from several repeats of the experiment for a single person (Figure 9). This is further evidence that the fluctuations are random and not due to some systematic effect.

Unlike some previous studies which used custom-built systems (e.g., [15,16,17]), we used a readily available commercial system that could easily be replicated, especially if the ERP part was omitted. Our results give reassurance to clinicians that the subject’s level of attention is not likely to affect measurements.

Another interesting source of variability was that shown by the time–frequency analysis using the MP method. Resonance modes of greatest amplitude came from different frequency bands (Figure 7B). These resonance modes were not suppressed in any systematic way, suggesting that broadband suppression values are probably better indicators of overall MOC function. Some previous studies have also shown that broadband suppression provided greater reliability than when a particular frequency band was used [36,37,38].

Despite the fact that the study showed no effect of change of modality on TEOAE suppression, it did provide another interesting result, namely that the amplitude of the P3 cognitive potential increased at the same time as normalized % TEOAE suppression decreased. It appears as if cortical processes may be able to compensate for some lack of synchronization at the peripheral level. This might explain the study by Marrufo-Perez et al. [39] who showed that adaptation to noise by cochlear implant users does not differ statistically from that of listeners with normal hearing. It is known that ERPs, as well as TEOAE suppression by CAS, show huge intersubject variability. In both cases, there are subjects with prominent responses and others in which it is difficult to record any, despite being normal in every other respect. In cases of ERPs, as with TEOAEs, such variability remains unexplained. Perhaps the variability is normal, and one system just compensates for the other. Such a compensation scheme might be generally small and not affect overall perception, but it might be crucial in cases of impairments. For example, the following hypothesis can be made: That cochlear implant users do not adapt to noise in any way different from normal hearing individuals. We suppose that although the MOC does not seem to be involved, it is really the cortex compensating for the MOC.

It is difficult to compare our results with previous studies, as there are few papers combining OAEs and ERPs. Furthermore, in these papers, the procedures vary significantly. For example, the recent study by Rao et al. [40] showed no connection between P3 and OAE suppression when listening in noise. The present results might be more closely related to a study by Dragicevic et al. [17] who showed some correlations in the modulation of OAEs and ERPs, although these were of a different kind than in the present study. There are also the interesting results by Riecke et al. [41] who showed some relationship between OAEs and the N1, although our work failed to detect such a connection.

There are some indications that attentional states may differentially affect MOC reflexes in the right and left ears [42]. However, in the current study, we decided not to complicate the issue and chose to record TEOAEs only from the right ear. Our reasoning was that TEOAE response levels are known to be higher in the right ear, and therefore SNRs would also be higher [43], and high SNRs are crucial for acquiring reliable MOC reflexes [19,20,21,38].

### 4.1. Limitations

One factor that can always have an effect on MOC measurements is middle ear muscle reflexes (MEMRs). Some researchers who use custom-built equipment have developed procedures that allow MEMR contamination of OAEs to be detected (e.g., [16,21]). Here, we used a system in which it was not possible to use such a procedure. Nevertheless, all ears had ART above 80 dB, which is much above the stimulation level of 65 dB used in TEOAE recordings (and the 60 dB for CAS). Together with earlier results which show that only a small percentage of ears are contaminated by MEMRs at such stimulus levels (e.g., [44,45]), we assume that MEMRs had little to no influence on the results reported here. In line with this idea, Francis et al. [16] used a specific test for MEMRs and reported that they found only a small fraction of single responses from each subject which were due to possible MEMR contamination. At the same time, they failed to find any systematic pattern in these responses (which they removed just to be safe) and concluded that they were unsure if they were indeed contaminated by MEMRs. This finding shows how difficult the MEMR problem is. Certainly, if MOC measures are to be used clinically it is necessary that commercial OAE systems provide ways of detecting MEMRs.

In our work, we decided not to do no-task recordings, as subjects became exhausted doing them. Furthermore, in our previous study [18], we did not find significant differences between OAEs recorded while performing a visual task and OAEs during the no-task condition. We also did not measure ERPs synchronized with visual stimuli. The reason was that we planned the experimental paradigm to demonstrate only that P3 could be evoked during an auditory task and would disappear when subjects were distracted by performing a visual task.

### 4.2. Implications

There seem to be no difference between the effect of visual and auditory attention on MOC as measured by TEOAEs. It seems that when attention is switched from one modality to another it is done by the cortex alone without the help of the periphery, in this case the ear. The study group was small (20 persons), although comparable with previous studies that showed a difference between auditory and visual tasks (e.g., [9,14,15]).

The ERP recordings were very important here. If they were not recorded, one could argue that there may have been no difference in the periphery (TEOAEs), since there would be no evidence the cortex was in a different state. The ERPs provide proof that there are indeed two different cortical effects of the performed tasks, while it seems there was no effect on TEOAEs. Furthermore, the negative correlation between suppression of TEOAEs and the P3 amplitude during the auditory task seems to suggest that the cortex compensates for lower synchronization at the MOC level.

A secondary aspect of this study, flowing from the primary finding that a change in attentional state does not seem to affect the MOC reflex, is that it is permissible to use distractors during suppression experiments. For example, it might be beneficial to show a movie while making MOC reflex measurements. This may help some patients, especially children, relax and sit still (e.g., [37,38]). At the same time, there seems to be no need to control for attention in OAE measurements (as suggested by other recent papers, e.g., [21]). Furthermore, if OAE suppression is not affected by conscious attention, one might conclude that sleep should not hinder the MOC inhibitory circuit. However, from our experience, when a person sleeps during an OAE recording, the noise levels increase, causing a decrease in SNR. The higher noise levels are mostly related to louder breathing and more uncontrolled movement. As already mentioned, high SNR is crucial for reliable MOC reflex testing, and therefore performing such measurements during sleep could be difficult.

A more general implication of this study is that making a connection between various aspects of sound processing and the MOC reflex needs to be done with caution. Even though we ensured good quality measurements with high SNRs, we did not find a difference between tasks (unlike in some previous experiments). This is in line with some other studies that failed to confirm earlier reported effects on the MOC reflex, for example, there are studies that have failed to show any connection between the MOC reflex and gender or laterality [46], adaptation to noise and central auditory processing [39,40], auditory processing disorders [47], tinnitus [48], or sickle cell disease [49]. Specifically, it has been suggested that previous work on auditory processing disorders probably did not fulfill appropriate SNR criteria in order to ensure reliability [47]. Nevertheless, despite the abovementioned findings, we do not completely discard the possibility that there is cooperation between ear and cortex when switching between modalities. However, such an effect on OAEs might be very small and below the level of detection with current group sizes, as Beim et al. also suggested [50].

## 5. Conclusions

The study showed there was no change in the MOC reflex when attention was switched from an auditory to a visual task. If any small change does exist it would need a large group and repeated measurements to reveal it. We conclude that there is no need for control of attention during auditory measurements. On the other hand, there does seem to be some connection between cortical attentional processes related to hearing and the MOC because there is a significant negative correlation between the OAE suppression level and amplitude of the P3 wave.
